# (1→3)-α-d-Glucooligosaccharides Increase the Killing Capacity of NK Cells against Selected Human Colon Cancer Cells

**DOI:** 10.3390/molecules28104212

**Published:** 2023-05-20

**Authors:** Marta Kinga Lemieszek, Paulina Adamczyk, Iwona Komaniecka, Wojciech Rzeski, Michał Tomczyk, Adrian Wiater

**Affiliations:** 1 Department of Medical Biology, Institute of Rural Health, ul. Jaczewskiego 2, 20-090 Lublin, Poland; wojciech.rzeski@mail.umcs.pl; 2 Department of Industrial and Environmental Microbiology, Institute of Biological Science, Maria Curie-Skłodowska University, ul. Akademicka 19, 20-033 Lublin, Poland; paulina.adamczyk@mail.umcs.pl; 3 Department of Genetics and Microbiology, Institute of Biological Science, Maria Curie-Skłodowska University, ul. Akademicka 19, 20-033 Lublin, Poland; iwona.komaniecka@mail.umcs.pl; 4 Department of Functional Anatomy and Cytobiology, Institute of Biological Science, Maria Curie-Skłodowska University, ul. Akademicka 19, 20-033 Lublin, Poland; 5 Department of Pharmacognosy, Faculty of Pharmacy with the Division of Laboratory Medicine, Medical University of Białystok, ul. Mickiewicza 2a, 15-230 Białystok, Poland; michal.tomczyk@umb.edu.pl

**Keywords:** (1→3)-α-d-glucooligosaccharides, immunomodulation, cancer immunotherapy, colon cancer, NK cells

## Abstract

Despite the progress of medicine, colorectal cancer has occupied one of the highest positions in the rankings of cancer morbidity and mortality for many years. Thus, alternative methods of its treatment are sought. One of the newer therapeutic strategies is immunotherapy based on NK cells (natural killers), which are the body’s first line of defense against cancer. The aim of the study was to verify the possibility of using (1→3)-α-d-glucooligosaccharides (GOSs) obtained via acid hydrolysis of (1→3)-α-d-glucan from the fruiting body of *Laetiporus sulphureus* to improve the anticancer effect of NK-92 cells, with proven clinical utility, against selected human colon adenocarcinoma cell lines LS180 and HT-29. The study revealed that the investigated oligosaccharides significantly enhanced the ability of NK-92 cells to eliminate the examined colon cancer cells, mostly by an increase in their cytotoxic activity. The most significant effect was observed in LS180 and HT-29 cells exposed to a two-times higher quantity of NK cells activated by 500 µg/mL GOS, wherein NK-92 killing properties increased for 20.5% (*p* < 0.001) and 24.8% (*p* < 0.001), respectively. The beneficial impact of (1→3)-α-d-glucooligosaccharides on the anticancer properties of NK-92 suggests their use in colon cancer immunotherapy as adjuvants; however, the obtained data require further investigation and confirmation.

## 1. Introduction

The latest data have revealed that colorectal cancer (CRC) is the third most common cancer and the second most fatal cancer in the world. In 2020, there were almost two million new cases of this type of cancer globally (1,931,590) and another million people (935,173) died of this disease [1]. These alarming statistics encourage further attempts to develop better strategies for the prevention and treatment of this type of cancer. One such strategy that has gained popularity and importance in recent years is immunotherapy, which is based on the mobilization and strengthening of the body’s own defense mechanisms to recognize and then effectively and safely eliminate cancerous cells. 

Given the key role of NK cells in fighting malignantly transformed cells, as well as clinical data correlating their amount with better survival in CRC patients [2,3], it seems prudent to base colon cancer therapy on an increase in the NK cell number, and first of all, enhancement of their function. NK cells are cytotoxic members of the large and plastic family of innate lymphoid cells [4] able to detect and rapidly kill abnormal cells without prior sensitization; thus, they are the first line of immune system defense against cells infected with bacteria and viruses as well as malignant cells [5]. The main challenge of oncotherapy based on NK cells is the source of the lymphocytes and the method of improving their anticancer activity. The present study especially focused on the latter issue and investigated the possibility of using (1→3)-α-d-glucooligosaccharides obtained via acid hydrolysis of (1→3)-α-d-glucan from the fruiting body of *Laetiporus sulphureus* to improve the cytotoxic effect of NK-92 cells (NK cells with the best documented anticancer activity in clinical studies) [6] against selected human colon adenocarcinoma cell lines LS180 and HT-29. 

For many years, (1→3)-α-d-glucans were thought to have no significant biological effects, mostly because they are insoluble in water. Nevertheless, given the presence of specific receptors on immune cells able to recognize glucan epitopes (destin-1, CR3, TLRs) [7,8], the possibility of their use as immune modifiers is increasingly being indicated, as confirmed in several scientific studies [9,10,11,12,13]; unfortunately, none of the cited studies directly involved NK cells. Considering the possibility of using (1→3)-α-d-glucans in immuno-oncology, it needs to be also highlighted that the anticancer effect of these compounds was previously described [14,15,16]; furthermore, there are also two scientific reports that revealed a beneficial effect of (1→3)-α-d-glucans in different colon cancer cell lines [17] and a murine model of colon cancer [12]. 

The breakdown of high-molecular-weight polysaccharides into low-molecular-weight oligosaccharides is important for improving their bioavailability, increasing the absorption of compounds by the organism, and fully utilizing the effectiveness of polysaccharides [18,19]. Oligosaccharides obtained by breaking down polysaccharides show anticancer properties, counteract oxidation, regulate immune responses, reduce inflammation, have neuroprotective and antimicrobial effects, lower lipid levels, reduce hypertension, inhibit obesity, lower blood sugar levels, and promote cell proliferation [20,21,22]. Due to these properties, oligosaccharides have been widely used in the food and pharmaceutical industries, as well as medicine [23].

In our previous studies, we reported the prebiotic activity of oligosaccharides preparation obtained by acid hydrolysis of (1→3)-α-d-glucan isolated from the fruiting bodies of the polypore fungus *L. sulphureus* [24]. Moreover, our preliminary research confirmed the modulating effect of (1→3)-α-d-glucooligosaccharides on the murine gut microbiota composition [25]. Nevertheless, revision of scientific data has revealed that there are no studies examining the influence of hydrolyzates of (1→3)-α-d-glucans on the ability of NK cells to eliminate colon cancer cells. Thus, this is the first study specially dedicated to this issue.

## 2. Results and Discussion

(1→3)-α-d-glucans are widespread in the mushroom world, especially in the cell walls of numerous representatives of the Basidiomycota class [26,27]. One of the highest contents of this group of molecules was detected in the cell walls of the fruiting bodies of *Laetiporus sulphureus* (Bull.: Fr.) Murrill (even 88%) [28]. This fact, as well as its common occurrence and tendency to form impressive colonies (several dozen fruiting bodies with a cap diameter of 10 to 50 cm and up to 45 kg weight) [29], prompted its choice as a source of glucooligosaccharides with potential immunomodulatory properties.

To estimate the average molecular mass, the crude water insoluble (1→3)-α-d-glucans isolated from the fruiting bodies of *L. sulphureus* were dissolved in 1 M NaOH and fractionated on a Sepharose CL-6B column, as was described elsewhere [30]. This analysis showed that the material was heterogeneous. The elution profile showed two main chromatographic peaks. The first one eluted very close to a void volume of the column, giving a molecular mass of about 1 mln Da (1000 kDa), and the second, broad chromatographic peak with the maximum at region corresponded to about 20–30 kDa (Figure S1).

**Figure 1:**
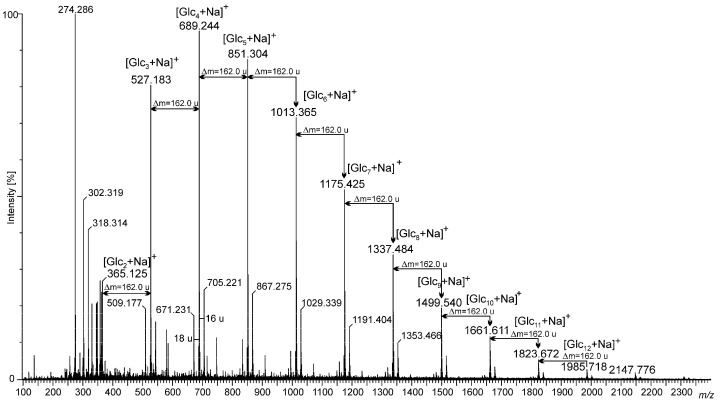
MALDI-TOF-MS spectrum in the positive ion mode of (1→3)-α-d-glucooligosaccharides obtained via the acid hydrolysis of (1→3)-α-d-glucan from the fruiting body of *Laetiporus sulphureus*.

The (1→3)-α-d-glucooligosaccharide preparations obtained by mild hydrolysis of *L. sulphureus* (1→3)-α-d-glucan using sulfuric acid were characterized by MALDI-TOF mass spectrometry. The mass spectrum obtained in the positive ion mode is shown in Figure 1. The hydrolyzate was composed of a wide spectrum of sodiated adducts of oligosaccharides containing from 2 to at least 12 glucose units. However, the main pool consisting of 3–7 glucose residues associated with a sodium ion ([Glc_x_ + Na]^+^) was represented by peaks at 527.183 *m*/*z* ([Glc_3_ + Na]^+^), 689.244 *m*/*z* ([Glc_4_ + Na]^+^), 851.304 *m*/*z* ([Glc_5_ + Na]^+^), 1013.365 *m*/*z* ([Glc_6_ + Na]^+^), and 1175.425 *m*/*z* ([Glc_7_ + Na]^+^), respectively. Small quantities of disaccharides (at 365.125 *m*/*z*) and oligomers containing from 8 to 12 glucose units (at 1337.484 *m*/*z*, 1499.540 *m*/*z*, 1661.611 *m*/*z*, 1823.672 *m*/*z*, and 1985.718 *m*/*z*), were also indicated. Furthermore, 2 very low-intensity signals derived from oligosaccharides composed of 13 and 14 glucose units were also observed in the region of 2150–2320 *m*/*z*. The most intense peaks of the spectrum were accompanied by small intense neighboring signals (see Figure 1). The mass difference of 18 u can be observed, derived from oligoglucose fragments deprived of water as a result of dehydration that occurred during acid hydrolysis. Moreover, each main signal corresponded to the oligosugar unit (e.g., sodiated tetrasaccharide at *m*/*z* 689.244, representing the B-type ion (B_4_^+^) according to the nomenclature proposed by Domon and Costello), was accompanied by a small intense peak larger by 16 u, also indicating the presence of C-type ions (in this case C_4_^+^) [31]. Signals visible in the region below 350 *m*/*z* were probably derived from the matrix used in MALDI-TOF analysis. 

**Figure 2:**
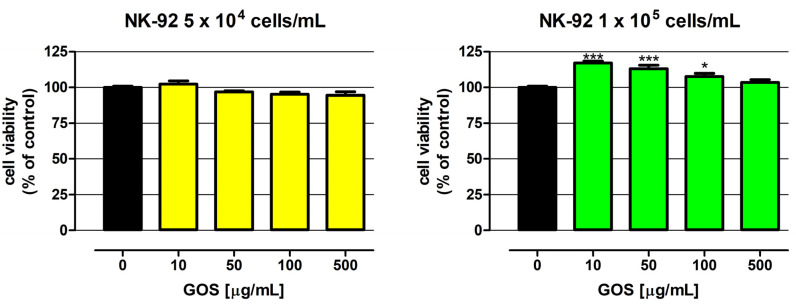
Influence of GOS on NK-92 cell viability. Cells were exposed to culture medium alone (control) or GOS at concentrations of 10, 50, 100, and 500 µg/mL for 48 h. Cell viability was determined by examination of metabolic activity using the MTT assay. Results are presented as mean ± SEM of at least six measurements. Statistically significant differences compared to the control at *p* < 0.05 (*), *p* < 0.001 (***). One-way ANOVA test; Dunnett’s post-hoc test.

In order to verify the usefulness of *L. sulphureus* (1→3)-α-d-glucooligosaccharides (GOSs) for immunotherapy of colon cancer, the direct impact of these molecules on the viability of NK-92 cells was examined in the first step. As presented in Figure 2, GOSs in the whole concentration range (10–500 µg/mL) did not have an impact on the viability of NK-92 cells at a density of 5 × 10^4^ cells/mL. On the contrary, in response to GOS applied at the concentrations of 10, 50, and 100 µg/mL, NK-92 cells at a density of 1 × 10^5^ cells/mL revealed an increase in metabolic activity by 17.1, 13.0, and 7.7%, respectively. Unfortunately, as mentioned before, there are no scientific data on the direct influence of α(1→3)-α-d-glucans on NK cell viability. Nevertheless, in vitro studies conducted by Bao et al. revealed that (1→3)-α-d-glucan isolated from the spores of *Ganoderma lucidum* after hydroxyethylation (PSG-HE-2; PSG-HE-3) and carboxymethylation (PSG-CM-1; PSG-CM-2; PSG-CM-3; PSG-CM-5; PSG-CM-7) significantly increased the proliferation of lymphocytes B and T. Additional studies conducted on mice proved the immunoenhancement properties of a glucan derivative, with a low degree of substitution with a carboxymethyl group (<0.28) and suitable solubility in water (PSG-CM-1), which significantly enhanced the T and B lymphocyte proliferation and antibody production [9]. The results obtained by Stephen-Victor et al. are also worth mentioning. Their study revealed that (1→3)-α-d-glucan isolated from *Aspergillus fumigatus* promoted activation of naive T cells and differentiation into Treg lymphocytes. The favorable influence on T cells discovered in the study, together with the reported beneficial effect of the investigated glucans on dendritic cell function, was the basis for the conclusion on the possibility of using (1→3)-α-d-glucan as adjuvants in vaccines against *A. fumigatus* infections [13].

**Figure 3:**
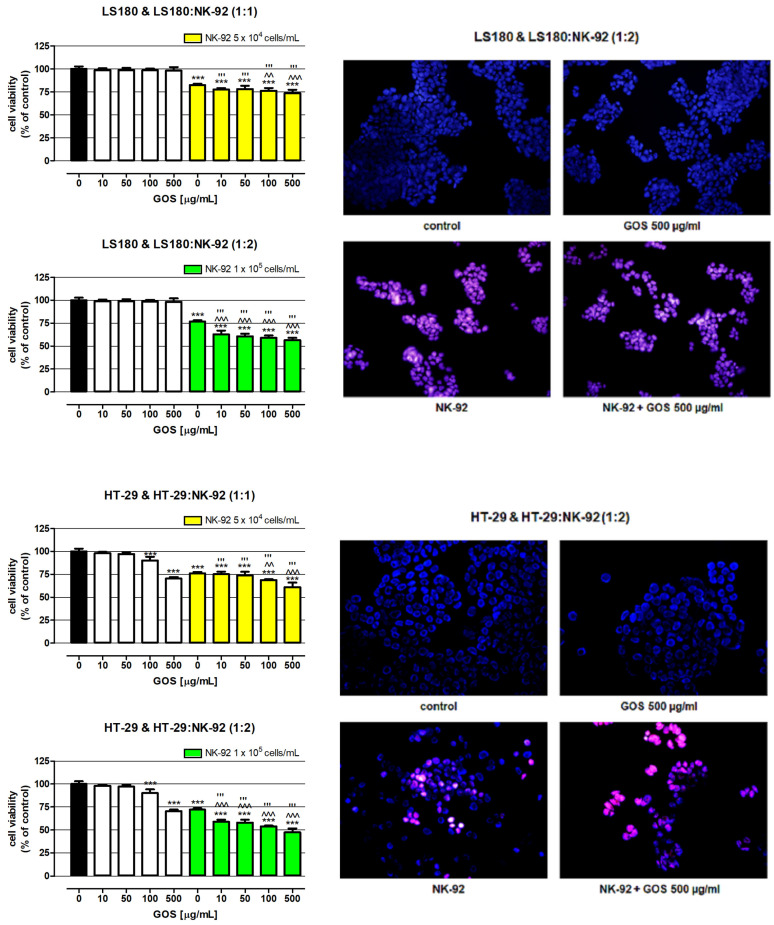
Influence of GOS on NK-92 cell cytotoxicity against human adenocarcinoma cell lines HT-29 and LS180. Cancer cells were exposed to culture medium alone (control) or GOS (10, 50, 100, and 500 µg/mL) used alone or NK-92 cells in the absence or presence of GOS for 48 h. NK-92 cells were used in the same or twofold higher concentration as the treated cancer cells. The viability of the investigated colon cancer cells in response to GOS used alone or together with the NK cells was determined by examination of cell metabolic activity using the MTT assay. Results are presented as mean ± SEM of at least five measurements. Statistically significant differences compared to the control at *p* < 0.001 (***). Statistically significant differences between cancer cells treated with GOS-activated NK-92 cells vs. cancer cells treated with only NK cells at *p* < 0.01 (^^), *p* < 0.001 (^^^). Statistically significant differences between cancer cells treated with GOS-activated NK-92 cells vs. cancer cells exposed to GOS at a corresponding concentration as that used for NK cell activation at *p* < 0.001 (’’’). One-way ANOVA test; Tukey’s post-hoc test. Cancer cell death in response to GOS as well as NK-92 cells activated by the tested compound was visualized by cell double staining with a mixture of propidium iodide and Hoechst 33342. The representative pictures from fluorescence microscopy are presented; the magnification is 200×.

In the next step of the study, the influence of GOS on NK-92 cytotoxicity against human adenocarcinoma cell lines HT-29 and LS180 was examined. First, the direct impact of GOS on the viability of colon cancer cells was determined. As presented in Figure 3, GOS applied in the whole concentration range did not have an impact on the metabolic activity of the LS180 cells, while the concentrations of 100 and 500 µg/mL significantly decreased the viability of the HT-29 cells by 9.92 and 29.57%, respectively. Initially, these results seemed slightly surprising in the light of results obtained by Czerwonka et al., who revealed significant antiproliferative properties of (1→3)-α-d-glucooligosaccharides (α-(1→3)-GOS) prepared from *Fomitopsis betulina* (1→3)-α-d-glucan against four different human colon cancer cell lines (HT-29, LS180, SW620, SW948). Nevertheless, the inhibition of colon cancer cell growth was observed after 96 h of treatment with α-(1→3)-GOS at concentrations ranging from 2.5 to 10 mg/mL [32]. Similar data were provided in a study conducted by Lavi et al. investigating polysaccharide extracts obtained from *Pleurotus pulmonarius* mycelium grown in liquid culture (ME) or α-glucan-rich *P. pulmonarius* fruiting bodies (FBE) or a mixture of both α-glucan and β-glucan. They found an evident decrease in the viability of several colon cancer cell lines (Caco-2, HT-29, HCT-116, HM-7, LS174T, RSB) after 48 and 60 h of exposure to both ME or FBS at concentrations of 0.5, 1, 2.5, and 5 mg/mL [17]. Noteworthy are also the results obtained by Masuda et al., who investigated α-glucan isolated from fruiting bodies of *Grifola frondose* (YM-2A) and reported its preventive and therapeutic effects in colon-26 tumor-bearing mice. The orally administered YM-2A (5 mg/mice daily; 5 days/week) inhibited colon cancer growth and increased the survival of the mice [12].

Additional studies strictly focused on NK cells showed that NK-92 cells used alone effectively killed colon cancer cells. NK cells at a density of 5 × 10^4^ cells/mL decreased the viability of LS180 and HT-29 cells by 17.62% and 23.82%, respectively. The observed beneficial effect of NK cells intensified with an increase in their number, reaching an additional 5.51% (LS180) and 3.96% (HT-29). The NK-92 cytotoxic effect was accelerated in the presence of GOS, depending on the oligosaccharide concentration and the number of effector cells. In co-cultures, wherein the ratio of cancer cells to lymphocytes was 1:1, the NK cytotoxic effect was effectively enhanced by GOS at concentrations of 100 and 500 µg/mL, lowering the viability of LS180 and HT-29 cells by 23.85%/31.22% and 26.37%/39.12, respectively. A twofold increase in the number of GOS-activated NK cells improved their anticancer effect, which was statistically significant in the whole range of oligosaccharide concentrations. The most effective elimination of colon cancer cells was observed in cultures treated with NK-92 cells activated by GOS at a concentration of 500 µg/mL, where the viability of LS180 and HT-29 cells was 56.43% and 47.43%, respectively. It should also be noted that doubling the number of NK cells slightly increased the elimination of cancer cells (increase by 5.5% in LS180 cells and by 4% in HT-29 cells). On the other hand, the comparison of data collected from co-cultures with a lower and higher density of NK-92 cells revealed that an anticancer effect of GOS-activated NK cells recorded in the second variant was on average 16.7% in LS180 cells and 15.1% in HT-29 cells, higher than in the first variant. The greater improvement in the killing capacity of NK cells by the tested substance observed in the culture of lymphocytes with a higher density may result from the increase in metabolic activity of lymphocytes in response to incubation with GOS, which was previously observed (Figure 2). Next to the examination of the metabolic activity of cancer cells, their response to NK-92 cells used alone or together with GOS was visualized using fluorescence microscopy. Performed studies have shown that GOS at a concentration of 500 µg/mL did not induce apoptosis or necrosis in investigated cancer cells; however, it significantly decreased the number of HT-29 cells, which could be explained by previously described alterations in the metabolism of cancer cells. NK-92 cells used alone effectively damaged the colon cancer cell membranes and this cytotoxic effect intensified in the presence of GOS. Furthermore, in LS180 cells treated with NK-92 cells used alone or together with GOS, apoptosis induction was also observed. In the case of HT-29 cells, programmed cell death was detected only in response to NK cells, while cancer cells treated with GOS-activated lymphocytes undergo necrosis. The observed enhancement of NK cells’ cancer-killing ability in response to GOS indicated its immuno-modulatory properties. It needs to be highlighted that a comparison of data collected from cancer cells exposed to GOS-activated NK cells with results obtained from cancer cells treated with the tested compound used alone (comparison of corresponding concentrations of GOS) revealed that the indirect anticancer effect of GOS (immunoenhancement effect; activation of NK cells) is significantly stronger than the direct impact. The obtained results agree with the aforementioned studies conducted by Masuda et al., who investigated the immunomodulatory properties of maitake α-glucan YM-2A in addition to its direct anticancer effect. In a murine colon-26 carcinoma model, they showed that orally administered YM-2A (5 mg/mice daily; 5 days/week) increased the amount of both CD4 and CD8 T cells expressing INF-γ in the spleen, elevated the number of INF-γ-releasing CD8 cells in tumor-draining lymph nodes, and increased the production of IL-12 by antigen-presenting cells, which all together enhanced the systemic immune response to cancer [12]. Interesting results were also provided by Murosaki et al., who examined the influence of nigerooligosaccharides (NOSs) obtained from *Acremonium* sp. S4G13 on the anticancer properties of murine hepatic mononuclear cells (MNCs). Their studies demonstrated that NOS at a concentration of 1 µg/mL significantly enhanced the MNC cytotoxic effect against mouse lymphoma cell line YAC-1. Moreover, additional in vivo studies indicated that a 1% solution of NOS administered orally to lymphoma-bearing mice significantly increased animal survival. Although Murosaki et al. suggested that the discovered anticancer effect of NOS was associated with enhancement of NK cell activity, potentially present in the tested murine hepatic mononuclear cells, their study did not provide convincing or even indirect evidence for this claim [10].

To the best of our knowledge, the presented results are the first scientific data indicating that *Laetiporus sulphureus* (1→3)-α-d-glucooligosaccharides have immunomodulatory properties associated with enhancement in the ability of NK cells to eliminate human colon cancer cells, mostly by an increase in their cytotoxic activity. It needs to be highlighted that a comparison of the anticancer effect of GOS-activated NK cells used in two different amounts revealed that increasing the number of lymphocytes did not have such a big impact on their killing abilities as their incubation with the tested compound, which in a culture with a higher NK cell density activated their antitumor activity more strongly. Although the present study was performed only in cell lines, their importance is associated with the use of NK-92 cells with proven therapeutic utility in clinical studies. The discovered beneficial effect of *L. sulphureus* (1→3)-α-d-glucooligosaccharides increases hope for its therapeutic use as an adjuvant in currently applied colon cancer immunotherapies.

## 3. Materials and Methods

### 3.1. Reagents

All chemicals were purchased from Sigma-Aldrich Chemical Co. (St. Louis, MO, USA) unless otherwise indicated.

### 3.2. Preparation of the (1→3)-α-d-Glucooligosaccharides (GOS)

The mixture of (1→3)-α-d-glucooligosaccharides was obtained by partial hydrolysis of (1→3)-α-d-glucan from the fruiting bodies of *Laetiporus sulphureus* in 0.1 M H_2_SO_4_ for 1 h at 100 °C, as previously described in Czerwonka et al. [32]. GOS obtained via acid hydrolysis of *L. sulphureus* (1→3)-α-d-glucan contained glucose (14.4%) and a mixture of (1→3)-α-d-glucooligosaccharides with a degree of polymerization (DP) from 2 to 9 (85.6%). The detailed chemical characteristic of GOS has been presented previously [24]. The stock solution of GOS was prepared by dissolving the lyophilizate in PBS (phosphate-buffered saline). The stock solution at a concentration of 100 mg/mL was stored at −80 °C. Working solutions of GOS were prepared by dissolving the stock solution in the culture medium.

### 3.3. Mass Spectrometry

MALDI-TOF-MS spectrometry was performed with a Waters Synapt G2-Si HDMS instrument (Waters Corporation, Milford, MA, USA) equipped with a 1 KHz Nd:YAG laser system (355 nm wavelength). Acquisition of the data was performed using MassLynx software, version 4.1 SCN916 (Waters Corporation, Wilmslow, UK). Mass spectra were assigned with multi-point external calibration using red phosphorous (Sigma-Aldrich, Saint Louis, MO, USA) in the range of 100–3000 Da. The sample was dissolved in water containing 0.1% TFA at a concentration of 20 µg/µL, subjected to ultrasonication for 5 min, and desalted on a piece of Parafilm^®^ with a few grains of Dowex 50WX8–100 (Serva, Heidelberg, Germany) converted into the H^+^ form. The desalted sample was mixed with a DHB matrix (2,5-dihydroxybenzoic acid (Sigma-Aldrich, Saint Louis, MO, USA), dissolved in 50% methanol, 20 µg/µL), and 1 µL of the mixture was transferred into the target plate wells. Spectra in the positive ion mode were recorded.

### 3.4. Cell Lines

Human natural killer cell line NK-92 was purchased from the American Type Culture Collection (ATCC, Manassas, VA, USA). Human colon adenocarcinoma cell lines HT-29 and LS180 were obtained from the European Collection of Cell Cultures (ECACC, Center for Applied Microbiology and Research, Salisbury, UK). The cells were cultured in Alpha Minimum Essential Medium with 2 mM L-glutamine and 1.5 g/L sodium bicarbonate supplemented with 0.2 mM inositol, 0.1 mM 2-mercaptoethanol, 0.02 mM folic acid, 200 U/mL recombinant IL-2, 12.5% horse serum, 12.5% fetal bovine serum, and a mixture of penicillin and streptomycin. The cells were maintained in a humidified atmosphere of 95% air and 5% CO_2_ at 37 °C.

### 3.5. MTT Assay

Basic variant: The cells were seeded on 96-well microplates at densities of 5 × 10^4^ cells/mL and 1 × 10^5^ cells/mL. Immediately after seeding on the microplates, the NK-92 cells were exposed to GOS at concentrations of 10, 50, 100, and 500 μg/mL. In contrast, the colon cancer cells were exposed to GOS at the above-indicated concentrations 24 h after cell seeding into the microplates.

Co-culture variant: The colon cancer cells were seeded on 96-well microplates at a density of 5 × 10^4^ cells/mL. The next day, the culture medium was removed and the cancer cells were exposed to NK-92 cells in the absence or presence of GOS at concentrations of 10, 50, 100, and 500 μg/mL. The proportion of colon cancer cells to NK cells was 1:1 or 1:2.

In both variants of the experiments, the cells were treated with GOS or NK-92 cells or GOS + NK-92 cells for 48 h; afterward, cell viability was examined by the MTT assay. In brief, the cells were incubated with the MTT solution (5 mg/mL in PBS) for 6 h. Then, formazan crystals were solubilized overnight in SDS buffer pH 7.4 (10% SDS in 0.01 N HCl), and the product was quantified spectrophotometrically by measuring the absorbance at the 570 nm wavelength using a microplate reader ELx800 (BioTek, Winooski, VT, USA).

### 3.6. Cell Death Differential Staining

Colon cancer cells were seeded on an 8-well chamber slide at densities 5 × 10^4^ cells/mL. The next day, the culture medium was removed and cells were treated with NK-92 cells at densities 1 × 10^5^ cells/mL in the absence or presence of the GOS at concentrations of 500 μg/mL. After 48 h of treatment, cancer cell death was examined using nuclear double staining, which differentiates apoptotic from necrotic cells. Cells were incubated for 5 min with a mixture of propidium iodide (0.15 mg/mL) and Hoechst 33342 (0.24 mg/mL). Then, stained cells were observed under a fluorescence microscope (Olympus BX51 System Microscope, Olympus Optical CO, Ltd., Tokyo, Japan). Cell images were captured using CellFamily AnalySIS software v 3.3 (Matrix Optics, Malaysia).

### 3.7. Statistical Analysis

The collected data were analyzed in the Microsoft Excel 2010 and GraphPad Prism 5.0 programs. The results were presented as the mean value and standard error of the mean (SEM). The data were analyzed by one-way ANOVA with Dunnett’s or Tukey’s post-hoc tests, and column statistics were used for comparisons. Significance was accepted at *p* < 0.05.

## Data Availability

Not applicable.

## Supplementary Materials

The following supporting information can be downloaded at: https://www.mdpi.com/article/10.3390/molecules28104212/s1, Figure S1: SEC chromatogram of the crude (1→3)-α-d-glucan isolated from the fruiting bodies of the polypore fungus *L. sulphureus*.

## Sample Availability

The sample obtained via the acid hydrolysis of (1→3)-α-d-glucan from the fruiting body of *Laetiporus sulphureus* ((1→3)-α-d-glucooligosaccharides) is available from the authors.
