# Supplementary material for: (1→3)-α-d-Glucooligosaccharides Increase the Killing Capacity of NK Cells against Selected Human Colon Cancer Cells
Source: Molecules. 2023 May 20;28(10):4212. doi: 10.3390/molecules28104212 (PMC10220765; doi:10.3390/molecules28104212)
Supplement: Supplementary file 1 [file molecules-28-04212-s001.zip › molecules-2383569-supplementary.pdf]

Supplementary Materials

(1→3)-*α*-D-Glucooligosaccharides increase the killing capacity of NK cells against selected human colon cancer cells

Marta Kinga Lemieszek, Paulina Adamczyk, Iwona Komaniecka, Wojciech Rzeski, Michał Tomczyk and Adrian Wiater

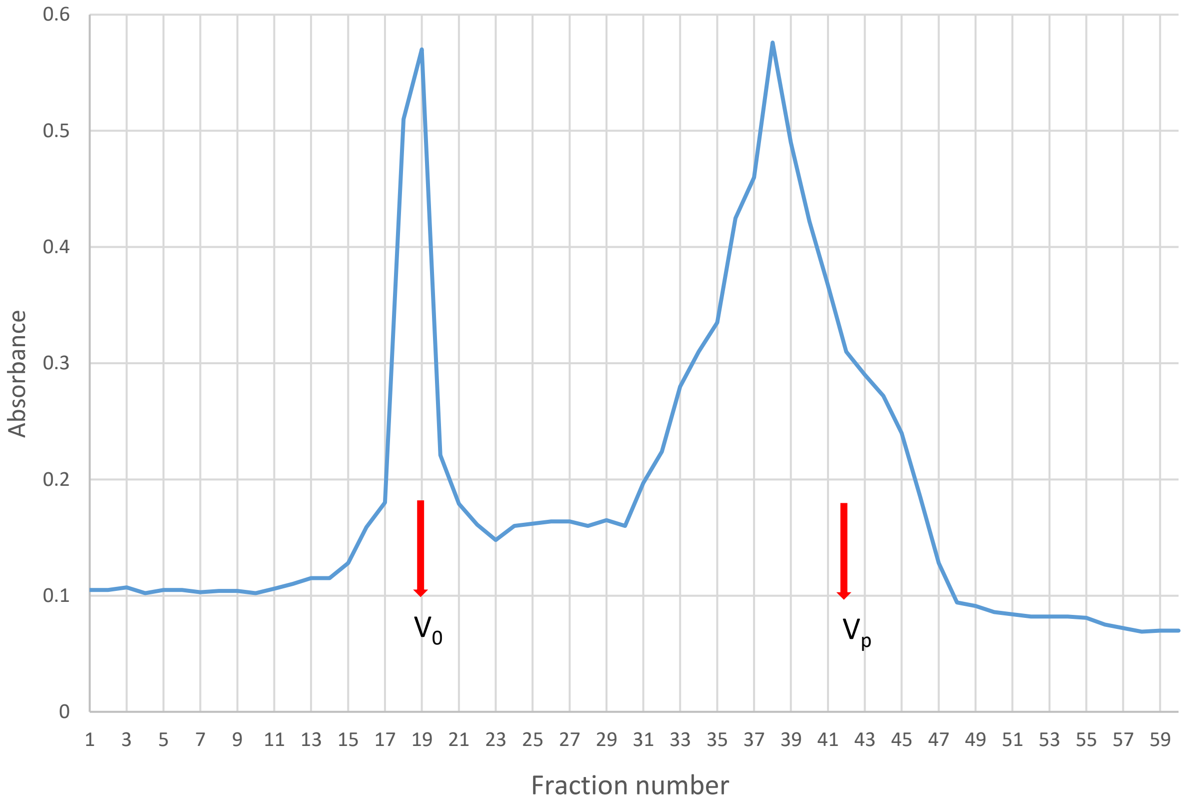

Figure S1. SEC chromatogram of the crude (1→3)-*α*-D-glucan isolated from the fruiting bodies of the polypore fungus *L. sulphureus* at Sepharose CL-6B column, eluted with 1 M NaOH, flow rate 0,2 mL/min, fraction size 1 mL. Column was calibrated using standards: Dextran Blue 2000 (Mw = 2 mln Da; Vo at 19 fraction) and Dextran 10 (Mw = 10 000 Da; Vp at 42 fraction) (both from Pharmacia, Uppsala, Sweden).
